# Training Volume and Training Frequency Changes Associated with Boston Marathon Race Performance

**DOI:** 10.1007/s40279-025-02304-4

**Published:** 2025-09-06

**Authors:** Alexandra F. DeJong Lempke, Kathryn E. Ackerman, Trent Stellingwerff, Louise M. Burke, Aaron L. Baggish, Pierre A. d’Hemecourt, Sophia Dyer, Chris Troyanos, Grace H. Saville, Kaya Adelzadeh, Bryan Holtzman, Anthony C. Hackney, Kristin E. Whitney

**Affiliations:** 1 Department of Physical Medicine and Rehabilitation, School of Medicine, Virginia Commonwealth University, 1223 East Marshall Street, Richmond, USA; 2 Institute for Women’s Health, Virginia Commonwealth University, Richmond, USA; 3 Women’s Health, Sports & Performance Institute, Boston, USA; 4 Neuroendocrine Unit, Massachusetts General Hospital, Harvard Medical School, Boston, USA; 5 Canadian Sport Institute Pacific, Victoria, Canada; 6 School of Exercise Science, Physical and Health Education, University of Victoria, Victoria, Canada; 7 Mary MacKillop Institute for Health Research, Australian Catholic University, Melbourne, Australia; 8 Department of Cardiology, Massachusetts General Hospital, Boston, USA; 9 Département Coeur-Caisseaux, Centre Hospitalier Universitaire Vaudois, Lausanne, Switzerland; 10 Harvard Medical School, Boston, USA; 11 Division of Sports Medicine, Boston Children’s Hospital, Boston, USA; 12 Boston University School of Medicine, Boston, USA; 13 Department of Emergency Medicine, Boston Medical Center, Boston, USA; 14 International Institute of Race Medicine, Plymouth, USA; 15 Sports Medicine Consultants, Plymouth, USA; 16 Exercise and Sport Science; Nutrition, University of North Carolina, Chapel Hill, USA

## Abstract

**Background:**

Physical training influences competitive marathon performance, including training volume and training frequency changes (TFCs) pre-race. Training intensity distribution (i.e., steady-state, quality sessions, interval training) and cross-training contribute to volume and TFCs that may influence performance.

**Objective:**

The aim of this study is to assess the relationships among training and TFCs preceding the 2022 Boston Marathon and race performance.

**Methods:**

Adult 2022 Boston Marathon registrants were contacted via email 1 month pre-race. Athletes reported demographics, training/racing experience, and training pre-race. TFCs were calculated by comparing two timeframes: 12–4 and 4–0 month pre-race training. Official race performance was obtained from chip timing data and demographics. Separate linear regressions were used to assess the effects of training and cross-training in 12–4 and 4–0 months pre-race and TFCs on performance, accounting for experience and demographics.

**Results:**

In total, 917 athletes were included (female: *n* = 495, 3:53 ± 0:37 h race times, 64.4 ± 24 km/week weekly distance; male: *n* = 422; 3:35 ± 0:39 h race times, 67.6 ± 26.2 km/week weekly distance). Higher running distance/week, running sessions/week (*n*), quality sessions/week (“hard sessions”; *n*), average distance in the 12–4 and 4–0 months pre-race (*p* ≤ 0.050), and more cross-training (*p* < 0.001) in the 4–0 months pre-race were associated with faster times and performance. Runners with TFCs of decreased running sessions/week (*p* = 0.035) had faster times and better performance versus athletes who maintained/increased volume.

**Conclusion:**

Habitually higher training exposure 12–4 and 4–0 months, but relatively reduced training frequency 4–0 months pre-race, contributed to better marathon performance.

**Supplementary Information:**

The online version contains supplementary material available at 10.1007/s40279-025-02304-4.

## Key Points

**Table:** 

This larger study aimed to assess variation in training frequency data up to 12 months prior to a marathon event in relationship to performance.
Faster race times were associated with higher overall training volumes, more speed-focused running sessions, more cross-training, and a relative reduction in running volume in the 4–0 months leading up to the race.
Athletes should be educated on the benefits of habitually high training exposures, but modulation of the training volume compared with their previous months of preparation to optimize performance.

## Introduction

Distance races have gained popularity worldwide over the last several decades, with an average one million runners competing in marathon events annually [1]. Large-scale marathons provide a unique opportunity to explore athletic performance and factors underpinning endurance running success. Multiple components impact endurance running performance [2, 3], including maximum oxygen uptake (V̇O_2_max; aerobic capacity) [2, 4–6], running economy [2, 6, 7], fractional utilization (percentage of V̇O_2_max during sustained exercise) [2], and physiological resilience (or fatigue resistance) [3]. One of the most instrumental factors influencing physiological adaptations is training frequencies/strategies in the months prior to competition [2, 4, 6, 8]. Historically, there has been a focus on increased overall preparatory training volume to achieve desired physiological adaptations. That is, increased habitual training volume at steady-state running paces is beneficial for improved endurance race performance [9]. Volume, however, does not account for intensity, structure, and periodization (i.e., tapering relative to habitual training) of endurance training programs, nor ancillary training (i.e., cross-training). While there is evidence supporting that overall training volume supports performance, training intensity distributions and time spent in these training zones influence distance running performance and are integral in popularized training plans [8]. These training components have the potential to influence physiological adaptations and subsequent competitive performance [10].

Deliberate training through interval-based running, quality/hard running sessions, and other high-intensity running workouts are often included in popularized training plans [8]. These induce different stimuli through aerobic and anaerobic pathways compared with steady-state running [11]. Previous assessments among world-class athletes identified that the inclusion and volume of tempo training and short-interval activities within overall training structures significantly positively influenced competitive performance, attributed, in part, to differential adaptations of the cardiovascular and neuromuscular systems [9]. Resistance training and other cross-training activities have also been found to improve running economy and endurance performance [12]. Evaluating multiple elements of training in preparation for a marathon running event may help to elucidate nuances in training frequencies and training frequency changes (TFCs) related to performance.

Popularized endurance training plans often implement a periodized approach, in which training is segmented into ordered phases leading up to competition to optimize peak preparedness on race day [8, 9, 13, 14]. Long-term training phases (macrocycles) are often labeled as “preparatory phases” consisting of the highest training volumes, with a transition toward higher-intensity training associated with improved race performance [8]. Then, the weeks leading up to the race (meso- to micro-cycles) employ a relatively reduced volume approach or “taper” while maintaining training intensity. While this general framework is widely employed, there are alternative approaches and philosophies utilized by athletes to enhance performance. There remains no consensus on which approach may be superior to promote athletic success [15]. These discrepancies are likely influenced by athlete experience/exposures, training age, and a range of sociodemographic factors [15]. Exploring short-term, pre-race TFCs from baseline training and their relationship to marathon performance is warranted.

The purpose of this study was to assess relationships among 2022 Boston Marathon race performance and running training, cross-training, and TFCs in months 12–4 versus months 4–0 pre-race. We hypothesized athletes with higher training volumes and intensities in 12–4 and 4–0 months pre-race and reduced training volume in 4–0 months (relative to 12–4 months) pre-race would have improved performance.

## Methods

### Study Design and Participants

This was a prospective observational cohort study, consisting of cross-sectional surveys and short-term prospective data collection to obtain race performance. Eligibility criteria included adults (ages ≥ 18 years) registered for the 2022 Boston Marathon. Registrants were recruited for participation through official host organization email correspondences sent weekly during the month pre-race. Interested individuals who met eligibility criteria completed informed consent and a brief pre-race survey through Research Electronic Data Capture (REDCap; Host: Harvard University) [16, 17]. The study was approved by the Harvard Medical School and Boston Children’s Hospital Institutional Review Board (IRB: no. IRB-P00041933). This study was performed in accordance with the ethical standards as laid down in the 1964 Declaration of Helsinki and its later amendments or comparable ethical standards. All data were de-identified and stored in an encrypted file accessible only by IRB-approved study personnel.

### Study Survey

The survey consisted of participant demographics and anthropometrics, training, and past medical history, as described previously (Table 1) [18]. Participants answered detailed questions regarding their running history, including years of dedicated marathon training and previous marathons (*n*). Participants also provided the following training information over the 12–4 and 4–0 months pre-race: weekly running and cross-training duration (hours), weekly sessions of running (*n*), weekly quality sessions (described in the survey as “running hard sessions”) (e.g., interval sessions, tempo runs, “fartleks,” or similar workouts; *n*), weekly cross-training sessions (*n*), and weekly running distance (in miles or kilometers [km], all converted to km) over the 12–4 and 4–0 months pre-race (Table 1). We intentionally selected 12–4 months for habitual training (macrocycle) and 4–0 month timeframes to represent recent training (mesocycle associated with tapering), consistently seen with training programs [19]. Participants consented to link survey data to official race times.

**Table 1:** Pre-race survey outcomes of interest and descriptions

Question category	Variable	Survey question	Response format
Personal information	Bib number	What is your 2022 Boston Marathon bib number?	*Open ended (numeric only)*
Demographics and anthropometrics	Age	What is your current age?	*Open ended (numeric only)*
	Height	What is your current height?	*Open ended (numeric only)*
	Weight	What is your current weight?	*Open ended (numeric only)*
	Race	What is your race?	*Select all that apply* Caucasian or white African American or Black Asian American or Pacific Islander American Indian or Alaskan Native More than one race Other
	Ethnicity	Are you of Hispanic or Latinx descent?	*Binary* Yes No
	Division	What division are you competing in at the 2022 Boston Marathon?	*Categorical* Male Male para-athlete Female Female para-athlete
Training and competition history	Years of marathon Training	How many years have you seriously pursued marathon training (e.g., focused training, coach, pursuit of performance)?	*Open ended*
	Number of past marathons	How many marathons have you completed in the past?	*Open ended*
Running training details	Weekly running duration	*[Over the last year]* AND *[Over the last 4 months]*, how many hours per week have you spent training (running only) on average? Please report weekly hours excluding strength & conditioning	*Categorical* 10–12.5 + h/week 7.5–10 h/week 5–7.5 h/week 2.5–5 h/week < 2.5 h/week
	Weekly running distance	*[Over the last year]* AND *[Over the last 4 months],* on average, how many miles or kilometers per week did you run?	*Open ended, with miles or kilometers option*
	Weekly running sessions	*[Over the last year]* AND *[Over the last 4 months]*, how many total runs did you do per week?	*Open ended*
	Weekly quality sessions	*[Over the last year]* AND *[Over the last 4 months]*, how many hard sessions (e.g., interval sessions, tempo runs, “fartlek” runs, or similar workouts by cross-training) did you do per week?	*Open ended*
Cross-training details	Weekly cross-training duration	*[Over the last year]* AND *[Over the last 4 months],* how many hours per week have you spent doing nonspecific general training for your sport (i.e., training outside of running [e.g., biking, swimming] or strength and conditioning)	*Categorical* > 10 h/week 7.5–10 h/week 5–7.5 h/week 2.5–5 h/week < 2.5 h/week
	Weekly cross-training sessions	*[Over the last year]* AND *[Over the last 4 months]*, how many total cross-training or strength and conditioning bouts did you do per week?	*Open ended*

The relevant subset of survey question details, including broad question categories, specific outcome variables of interest, question wording, and response types, are provided. Answer banks were provided for questions with categorical outcomes. Questions pertaining to running and cross-training behaviors were repeated twice for behaviors in the past year (12-4 months), and behaviors in the last 4 months (4-0 months).

### Data Processing

Incomplete data entries and those who did not complete the 2022 Boston Marathon were removed from analyses. Official race times, sex, and age were used to calculate World Athletics (WA) points [20]. WA points were subsequently used to determine performance tiering using the cut-off criteria presented by McKay et al. in 2021, with average personal best marathon times in the past 3 years by tier [21].

TFCs were calculated to determine the relative change in the number of weekly running, quality, and cross-training sessions. Training frequencies in the 12–4 months were subtracted from training frequencies in the 4–0 months to determine whether runners modified their training leading up to the race as follows:

$$\mathrm{TMCs}=\left(\text{Avg weekly activities} 4-0 \mathrm{months}-\text{Avg weekly activities} 12-4 \mathrm{months}\right).$$

### Statistical Analyses

Descriptive statistics were used to assess participant demographics, anthropometrics, training frequencies, TFCs, and performance. Independent *t*-tests were used to compare mean training outcomes by sex. Official race times in hours and minutes were converted to fractions of hours for analyses. Variables obtained as categorical through the survey set-up (running hours/week, cross-training hours/week) were reported as counts and percentages. Continuous variables (weekly running distance as well as running, quality, and cross-training sessions) were assessed for normality and then reported as mean ± standard deviation. Separate multivariate linear regression models were used to assess the effects of training and cross-training outcomes 12–4 (model 1) and 4–0 (model 2) months pre-race, and the effects of TFCs (model 3), on official race times and WA points. The interaction between weekly running and cross-training sessions was included to determine overall weekly activities. Age, sex, self-reported years of marathon-specific training, and number of previous marathons representing running experience were included as model covariates. Reference levels for post hoc pairwise comparisons for training hours were set at > 10 h/week. Alpha was set a priori to 0.05 for all analyses. Statistical analyses were conducted using R (v 2023.03.0 + 386) and Jamovi (v2.3.18.0).

## Results

**Fig. 1:**
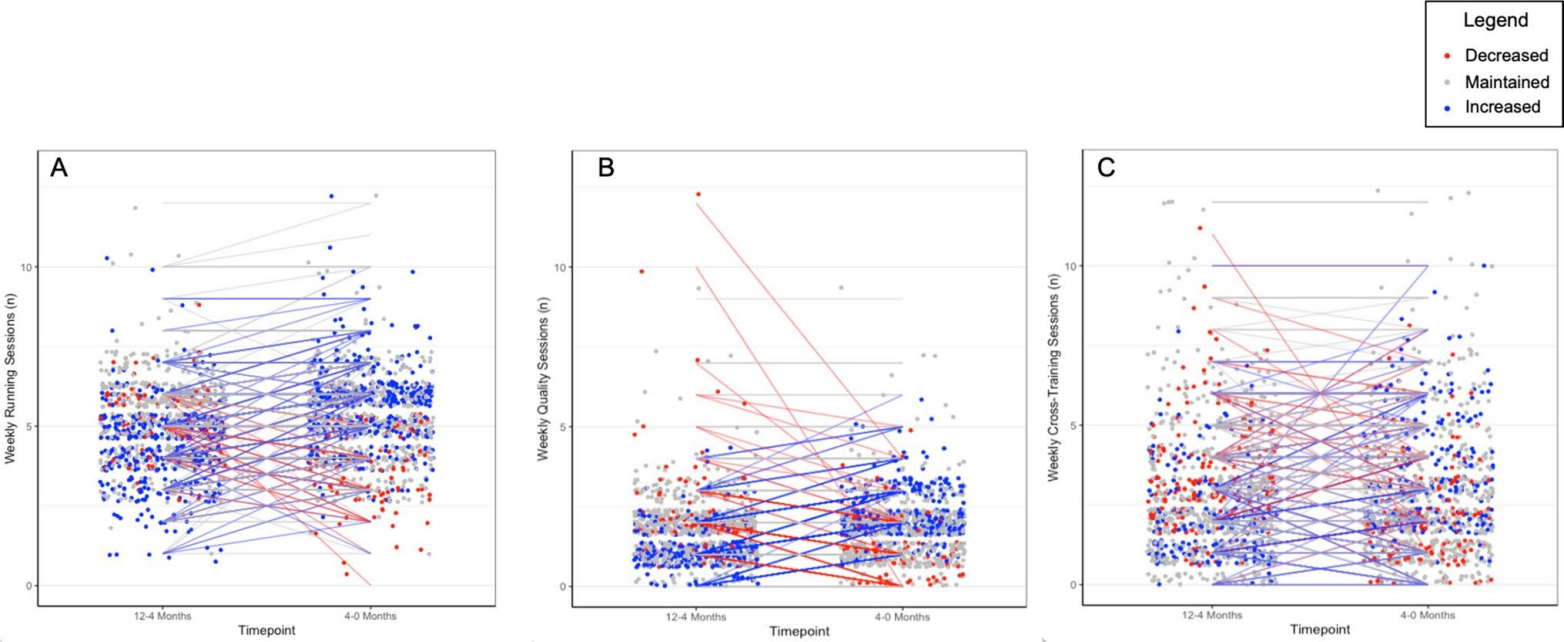
**A**–**C** Training frequency changes between 12 and 4 and 4 and 0 months pre-race for **A** weekly running sessions, **B** weekly quality sessions, and **C** weekly cross-training sessions. Each individual point represents a participant, with red points representing individuals who decreased activity, grey who maintained activity, and blue who increased activity. The lines using the same color scheme as the points represent direction of change based on starting number of activities

Of 1063 2022 Boston Marathon athletes enrolled, 917 had complete survey and training responses, representing 4% of the 2022 Marathon field (female: *n* = 495 [5% female field]; male: *n* = 422 [3% male field]; 95% tier 2 [trained/developmental]; Table 2). Athletes reported an average of 9 ± 8 years of dedicated marathon training and an average of 16 ± 26 previous marathons. Average reported training by sex and overall is provided in Table 2, with TFCs in Fig. 1.

**Table 2:** Participant demographics, running experience, and training details of included study participants

Outcome variable	Females (*n* = 495) *5% of female field size*	Males (*n* = 422) *3% of male field size*	All Runners (*n* = 917) *4% of total field size*
Age (years)	43 ± 13	52 ± 13	47 ± 14
BMI (kg/m^2^)	21.2 ± 3.0	22.8 ± 2.9	21.9 ± 3.1
Race (%)	Caucasian or White: 92% Asian Am. or Pacific Islander: 5% Black or AA: 1% More than one race: 1% Other: 1%	Caucasian or White: 92% Asian Am. or Pacific Islander: 5% Black or AA: < 1% More than one race: < 1% Other: 2%	Caucasian or White: 92% Asian Am. or Pacific Islander: 5% Black or AA: 1% More than one race: 1% Other: 1%
Ethnicity (%)	Non-Hispanic/ Latino: 93% Hispanic/Latino: 7%	Non-Hispanic/ Latino: 93% Hispanic/Latino: 7%	Non-Hispanic/ Latino: 93% Hispanic/Latino: 7%
Country of residence	North America: 93% (86% USA; 6% Canada; < 1% Mexico, Costa Rica, and DR) European: 5% (3% UK; < 1% each: Albania, Croatia, Finland, France, Germany, Greece, Ireland, Italy, Netherlands, Norway, Spain, Sweden, and Switzerland) South American: 1% (< 1% each: Argentina, Brazil, Colombia, and Ecuador) Asian: < 1% (< 1% each: Hong Kong, India, S. Korea, and Philippines) African: < 1% (< 1% each: Kenya and S. Africa) Australian/Oceanian: < 1% (< 1% each: Australia, New Zealand)	North America: 92% (84% USA; 7% Canada; 1% Mexico and Costa Rica; < 1% DR) European: 6% (3% UK; < 1% each: Albania, Croatia, Finland, France, Germany, Greece, Ireland, Italy, Netherlands, Norway, Spain, Sweden, and Switzerland) South American: 1% (< 1% each: Argentina, Brazil, Colombia, and Ecuador) Asian: < 1% (< 1% each: Hong Kong, India, S. Korea, and Philippines) African: < 1% (< 1% each: Kenya and S. Africa) Australian/Oceanian: < 1% (< 1% each: Australia and New Zealand)	North America: 92% (84% USA; 7% Canada; 1% Mexico and Costa Rica; < 1% DR) European: 6% (3% UK; < 1% each: Albania, Croatia, Finland, France, Germany, Greece, Ireland, Italy, Netherlands, Norway, Spain, Sweden, and Switzerland) South American: 1% (< 1% each: Argentina, Brazil, Colombia, and Ecuador) Asian: < 1% (< 1% each: Hong Kong, India, S. Korea, and Philippines) African: < 1% (< 1% each: Kenya and S. Africa) Australian/Oceanian: < 1% (< 1% each: Australia and New Zealand)
Dedicated running training (years)^a^	8 ± 7	10 ± 9	9 ± 8
Previous marathons (*n*)^a^	13 ± 25	19 ± 26	16 ± 26
Training and performance classification and 3-year average personal best marathon time: Tier 5 World Class (≥ 1158 WA points) Tier 4 Elite/International (986–1157 WA points) Tier 3 Highly Trained/National (742–985 WA points) Tier 2 Trained/Developmental (≤ 742 WA points)	Tier 5: 0% Tier 4: 0% Tier 3: 8% (3:03 ± 0:09) Tier 2: 92% (3:42 ± 0:30)	Tier 5: 0% Tier 4: 0% Tier 3: 2% (2:28 ± 0:10) Tier 2: 98% (3:21 ± 0:29)	Tier 5: 0% Tier 4: 0% Tier 3: 5% (3:00 ± 0:14) Tier 2: 95% (3:32 ± 0:32)
Official race times (hours:mins; decimal values)^a^	3:53 ± 0:37 (3.89 ± 0.61)	3:35 ± 0:39 (3.58 ± 0.65)	3:45 ± 0:39 (3.75 ± 0.65)
WA points^a^	485 ± 202	256 ± 206	378 ± 233
Weekly running duration (hour bins)^a^ *12–4 months only*	> 10 h: 25% 7.5–10 h: 33% 5–7.5 h: 34% 2.5–5 h: 8% < 2.5 h: < 1%	> 10 h: 15% 7.5–10 h: 34% 5–7.5 h: 36% 2.5–5 h: 14% < 2.5 h: 1%	> 10 h: 20% 7.5–10 h: 34% 5–7.5 h: 35% 2.5–5 h: 11% < 2.5 h: < 1%
Weekly running distance (km/week)^b^ *12–4 months only*	64.4 ± 24.2	67.6 ± 26.6	66.1 ± 25.2
Weekly running sessions (*n*)	12–4 months: 4.95 ± 1.44 4–0 months: 5.22 ± 1.38 + 5.5% change	12–4 months: 4.96 ± 1.41 4–0 months: 5.32 ± 1.47 + 7.3% change	12–4 months: 4.95 ± 1.42 4–0 months: 5.26 ± 1.42 + 6.3% change
Weekly quality sessions (*n*)	12–4 months: 1.63 ± 1.05 4–0 months: 1.84 ± 1.06 + 12.9% change	12–4 months: 1.68 ± 1.09 4–0 months: 1.83 ± 0.94 + 8.9% change	12–4 months: 1.65 ± 1.07 4–0 months: 1.83 ± 1.00 + 10.9% change
Weekly cross-training duration (hour bins)^b^ *12–4 months only*	> 10 h: 8% 7.5–10 h: 5% 5–7.5 h: 15% 2.5–5 h: 32% < 2.5 h: 40%	> 10 h: 6% 7.5–10 h: 5% 5–7.5 h: 11% 2.5–5 h: 32% < 2.5 h: 46%	> 10 h: 7% 7.5–10 h: 5% 5–7.5 h: 13% 2.5–5 h: 32% < 2.5 h: 43%
Weekly cross-training sessions (*n*)^b,c^	12–4 months: 2.90 ± 2.05 4–0 months: 2.95 ± 1.93 + 1.7% change	12–4 months: 2.44 ± 2.03 4–0 months: 2.41 ± 2.03 − 1.2% change	12–4 months: 2.68 ± 2.05 4–0 months: 2.70 ± 1.99 + 0.8% change
Weekly running sessions + weekly cross-training sessions (*n*)^b,c^	12–4 months: 7.85 ± 2.41 4–0 months: 8.16 ± 2.24 + 3.9% change	12–4 months: 7.40 ± 2.44 4–0 months: 7.73 ± 2.44 + 4.5% change	12–4 months: 7.64 ± 2.43 4–0 months: 7.96 ± 2.34 + 4.2% change

*Am.* American, *AA* African American, *DR* Dominican Republic, *UK* United Kingdom, *S.* South, *WA* World Athletics

^a^Statistical significance at *p* ≤ 0.05 between females and males

^b^Statistical significance at *p* < 0.05 between females and males for 12–4 months

^c^Statistical significance at *p* < 0.05 between females and males for 4–0 months

### Training Frequencies 12–4 Months Pre-race

Accounting for running experience, training and cross-training frequencies 12–4 months pre-race explained 48.5% of the variance in official race times, and 63.0% in WA points (*R*^2^ = 0.485, 0.630; *p* < 0.001; Table 3; Electronic Supplemental Materials [ESM] Table S1). Weekly running duration, running and quality sessions, and running distance 12–4 months pre-race were significant predictors (*p* ≤ 0.050). Running > 10 h/week 12–4 months pre-race was associated with improved race times compared with lesser time categories (*β* estimate [95% confidence interval {CI}] range − 0.22 [− 0.37, − 0.08] to –0.09 [− 0.21, 0.01]; *p* ≤ 0.050). For each additional running session/week in the 12–4 months pre-race, participants improved their race time by ~ 3.6 min (*β* [95% CI] − 0.06 [− 0.13, − 0.02]; *p* = 0.010) or 27.4 WA points (*β* [95% CI] 27.4 [10.3, 44.4]; *p* = 0.002). With each additional quality session/week in the 12–4 months pre-race, participants improved their race time by ~ 16.2 min (*β* [95% CI] − 0.27 [− 0.42, − 0.10]; *p* < 0.001) or 73.6 points (*β* [95% CI] 73.6 [24.6, 122.6]; *p* = 0.003). For every 1 km increase in running distance/week in the 12–4 months pre-race, participants improved their race time by ~ 0.6 min (*β* [95% CI] − 0.01 [− 0.02, − 0.005]; *p* < 0.001) or 2.4 points (*β* [95% CI] 2.4 [1.6, 3.3]; *p* < 0.001). No other training predictors 12–4 months pre-race were statistically significant. Age and sex were significant covariates (*β* [95% CI] range − 0.41 [− 0.48, − 0.34] to 0.02 [0.01, 0.03]; *p* ≤ 0.050), while running experience covariates were not (Table 3; ESM Table S1).

**Table 3:** Relationships between training behaviors in the 12–4 months pre-race and marathon performance, accounting for age, sex, and running experience

Model component	Outcome variable	Comparison	*β* estimate (95% confidence interval)	*t* statistic	*p*-value
Predictors *(overall model fit: R*^2^ = *0.485*, *p* < *0.001)*	Weekly running duration^a^	> 10 versus 7.5–10 h	− 0.09 (− 0.21, 0.01)	− 1.7	0.076
		> 10 versus 5–7.5 h	− 0.19 (− 0.30, − 0.08)	− 3.3	0.001^a^
		> 10 versus 2.5–5 h	− 0.22 (− 0.37, − 0.08)	− 2.9	0.003^a^
		> 10 versus < 2.5 h	− 0.12 (− 0.24, − 0.01)	− 2.1	0.044^a^
	Weekly running distance^a^	*Continuous*	− 0.01 (− 0.02, − 0.005)	− 5.3	< 0.001^a^
	Weekly running sessions^a^	*Continuous*	− 0.06 (− 0.13, − 0.02)	− 2.5	0.010^a^
	Weekly quality sessions^a^	*Continuous*	− 0.27 (− 0.42, − 0.10)	− 3.4	< 0.001^a^
	Weekly cross − training duration	> 10 versus 7.5–10 h	− 0.07 (− 0.29, 0.14)	− 0.6	0.518
		> 10 versus 5–7.5 h	− 0.03 (− 0.22, 0.16)	− 0.3	0.749
		> 10 versus 2.5–5 h	− 0.15 (− 0.33, 0.03)	− 1.6	0.106
		> 10 versus < 2.5 h	− 0.13 (− 0.32, 0.06)	− 1.4	0.179
	Weekly cross − training sessions	*Continuous*	− 0.01 (− 0.10, 0.01)	− 1.0	0.329
	Weekly running sessions and weekly cross − training sessions	*Continuous*	− 0.01 (− 0.04, 0.02)	− 1.3	0.192
Covariates	Number of previous marathons	*Continuous*	< − 0.01 (− 0.002, < 0.001)	− 0.9	0.392
	Years of marathon training	*Continuous*	0.01 (0.001, 0.01)	− 1.0	0.341
	Age^a^	*Continuous*	0.02 (0.01, 0.03)	15.2	< 0.001^a^
	Sex^a^	*Male versus female participants*	− 0.41 (− 0.48, − 0.34)	− 12.3	< 0.001^a^

Linear regression assessing the influence of training behaviors in the 12–4 months pre-race for the 2022 Boston Marathon on official race times

^a^Signifies statistical significance at *p* ≤ 0.050

### Training Frequencies 4–0 Months Pre-race

Accounting for running experience, training, and cross-training frequencies 4–0 months pre-race explained 49.0% of the variance in official race times, and 63.3% in WA points (*R*^2^ = 0.490, 0.633; *p* < 0.001; Table 4; ESM Table S2). Weekly running duration, running and quality sessions, running distance, cross-training, and the interaction between weekly running and cross-training 4–0 months pre-race were significant predictors of performance (*p* ≤ 0.050). Running > 10 h/week 4–0 months pre-race was significantly associated with improved marathon performance compared with other time categories (*β* [95% CI] range  − 0.21 [− 0.54, 0.13] to − 0.11 [− 0.21, − 0.003]; *p* ≤ 0.050). For each additional running session/week 4–0 months pre-race, participants improved their race time by an average of 3 min (*β* [95% CI] − 0.05 [− 0.10, − 0.01]; *p* = 0.023) or 9.5 WA points (*β* [95% CI] 9.5 [2.5, 38.9]; *p* < 0.001). With each additional quality session/week, participants improved their race time by ~ 17 min (*β* [95% CI] − 0.29 [− 0.43, − 0.16]; *p* < 0.001) or 76.6 points (*β* [95% CI] 76.6 [34.5, 115.0]; *p* < 0.001). For every 1 km increase in running distance/week 4–0 months pre-race, participants improved their official race time by ~ 13 min (*β* [95% CI] − 0.22 [− 0.48, − 0.34]; *p* < 0.001) or 2.9 points (*β* [95% CI] 2.9 [2.2, 3.5]; *p* < 0.001). For every 1-day increase in cross-training sessions/week in the 4–0 months pre-race, participants improved their official race times by ~ 6 min (*β* [95% CI] − 0.10 [− 0.16, − 0.04]; *p* < 0.001) or 7.2 points (*β* [95% CI] 7.2 [1.4, 25.3]; *p* = 0.023). Participants with combined increased running sessions/week and cross-training sessions/week in the 4–0 months pre-race improved their race times by ~ 1.2 min (*β* [95% CI] − 0.02 [− 0.03, − 0.01]; *p* = 0.004) or 4.8 points (*β* [95% CI] 4.8 [1.6, 8.1]; *p* < 0.001). Age and sex were significant covariates (*β* [95% CI] range − 0.41 [− 0.48, − 0.35] to 0.02 [− 0.02, 0.03]; *p* ≤ 0.050), while running experience covariates were not (Table 4; ESM Table S2).

**Table 4:** Relationships between training behaviors in the 4–0 months pre-race and marathon performance, accounting for age, sex, and running experience

Model component	Outcome variable	Comparison	*β* estimate (95% confidence interval)	*t* statistic	*p*-value
Predictors *(overall model fit: R*^*2*^ = *0.490, p* < *0.001)*	Weekly running duration^a^	> 10 versus 7.5–10 h	− 0.11 (− 0.21, − 0.003)	− 2.0	0.049^a^
		> 10 versus 5–7.5 h	− 0.18 (− 0.29, − 0.07)	− 3.2	0.001^a^
		> 10 versus 2.5–5 h	− 0.18 (− 0.32, − 0.04)	− 2.5	0.008^a^
		> 10 versus < 2.5 h	− 0.21 (− 0.54, 0.13)	− 2.6	0.075
	Weekly running distance^a^	*Continuous*	− 0.22 (− 0.48, − 0.34)	− 8.6	< 0.001^a^
	Weekly running sessions^a^	*Continuous*	− 0.05 (− 0.10, − 0.01)	− 2.3	0.023^a^
	Weekly quality sessions^a^	*Continuous*	− 0.29 (− 0.43, − 0.16)	− 4.4	< 0.001^a^
	Weekly cross-training duration	> 10 versus 7.5–10 h	− 0.09 (− 0.30, 0.13)	− 1.0	0.415
		> 10 versus 5–7.5 h	− 0.05 (− 0.23, 0.14)	0.5	0.612
		> 10 versus 2.5–5 h	− 0.19 (− 0.43, 0.07)	− 1.1	0.325
		> 10 versus < 2.5 h	− 0.17 (− 0.36, 0.01)	− 1.60	0.063
	Weekly cross-training sessions	*Continuous*	− 0.10 (− 0.16, − 0.04)	− 3.5	< 0.001^a^
	Weekly running sessions and weekly cross-training sessions	*Continuous*	− 0.02 (− 0.03, − 0.01)	− 2.9	0.004^a^
Covariates	Number of previous marathons	*Continuous*	− 0.10 (− 0.002, < 0.001)	− 0.1	0.934
	Years of marathon training	*Continuous*	− 0.70 (− 0.90, 0.10)	− 0.2	0.875
	Age^a^	*Continuous*	0.02 (− 0.02, 0.03)	15.2	< 0.001^a^
	Sex^a^	*Male versus female participants*	− 0.41 (− 0.48, − 0.35)	− 12.3	< 0.001^a^

Linear regression assessing the influence of training behaviors in the 4–0 months pre-race for the 2022 Boston Marathon on official race times

^a^Signifies statistical significance at *p* ≤ 0.050

### Training Frequency Changes 12–4 and 4–0 Months Pre-race

Accounting for running experience, TFCs 4–0 months pre-race compared with 12–4 months explained 36.6% of the variance in official race times, and 52.6% in WA points (*R*^2^ = 0.366, 0.526; *p* < 0.001; Table 5; ESM Table S3). The number of weekly runs was a significant predictor of race performance (*p* < 0.050). Participants who decreased their number of weekly running sessions in 4–0 months had faster official race times by an average of 3 min (*β* [95% CI] − 0.05 [− 0.09, − 0.003]; *p* = 0.035) or 14.6 WA points (*β* [95% CI] 14.6 [1.7, 27.5]; *p* = 0.026). Age and sex were significant covariates (*β* [95% CI] range − 0.58 [− 0.65, − 0.50] to 0.03 [0.02, 0.05]; *p* < 0.001).

**Table 5:** Relationships between training frequency changes from 12–4 to 4–0 months pre-race and marathon performance, accounting for age, sex, and running experience

Model component	Outcome variable (*F*-statistic, *p*-value)	Comparison	*β* estimate (95% confidence interval)	*t* statistic	*p*-value
Predictors *(overall model fit: R*^*2*^ = *0.366, p* < *0.001)*	Δ Weekly running sessions^a^	*Continuous*	− 0.05 (− 0.09, − 0.003)	− 2.1	0.035^a^
	Δ Weekly quality sessions	*Continuous*	− 0.01 (− 0.03, 0.04)	− 0.4	0.720
	Δ Weekly cross-training sessions	*Continuous*	− 0.01 (− 0.04, 0.01)	− 1.0	0.322
Covariates	Number of previous marathons	*Continuous*	− 0.01 (− 0.02, 0.002)	− 0.2	0.826
	Years of marathon training	*Continuous*	< − 0.01 (− 0.009, < 0.01)	− 1.9	0.067
	Age^a^	*Continuous*	− 0.58 (− 0.65, − 0.50)	17.7	< 0.001^a^
	Sex^a^	*Male versus female participants*	0.03 (0.02, 0.05)	16.2	< 0.001^a^

Linear regression assessing the influence of training behavior changes from the 12–4 to 4–0 months leading up to the 2022 Boston Marathon on race performance through official race times

^a^Signifies statistical significance at *p* ≤ 0.050

## Discussion

This study aimed to assess the relationships among race performance and training frequencies and TFCs in a large-scale marathon event. Increased running training volume and quality sessions in the 12–4 and 4–0 months pre-race and a higher number of cross-training sessions in 4–0 months pre-race were associated with improved performance. Importantly, reducing training frequency of weekly running sessions in 4–0 versus 12–4 months pre-race was associated with improved performance. These findings have key implications for athlete education for competitive endurance running preparation, indicating that a relative reduction in training volume pre-race may improve race performance, particularly for male participants given noted sex-based differences in training volumes and significant sex covariates across analyses.

### Pre-race Training Frequencies

Our primary hypotheses were supported; higher weekly running training exposure through the volume of running and quality sessions and cross-training 4–0 months pre-race were associated with better performance. Additionally, a higher running training volume 12–4 months pre-race was associated with better performance. Broad assessments of running distance and time were intended to provide surrogate measures of training loading exposure, while the number of quality sessions was intended to provide surrogate measures of running intensity and training variety in the context of an overall training regimen [9, 22]. The present findings corroborate the findings of Casado et al., who found that elite athletes who conducted more steady-state distance running along with training variety through quality sessions recorded the best competitive endurance race times [9]. While our findings are in alignment with previous literature, we were unable to examine training intensity distributions such as the three-tiered model in which activities are categorized as low-, moderate-, and high-intensity and which is often used in popularized training plans [8]. For example, polarized training plans incorporate a large volume of low-intensity with more high-intensity and minimal moderate-intensity training, while pyramidal training plans incorporate a large volume of low-intensity training, some moderate-intensity training, and less high-intensity training [8]. Unfortunately, it was beyond the capabilities of the present study to examine exact training approaches or training intensity distributions on the basis of the survey set-up, but these should be considered in the overall training programming in relationship to endurance performance in future investigations.

Our findings demonstrating performance benefits of overall higher volume are consistent with training principles underpinning popularized training programs [23–25] that often emphasize building running exposure during preparatory race phases followed by a brief, modest pre-race reduction in volume, such as in a classical (linear) periodization plan as opposed to reverse periodization that increases volume with decreased intensity over time [8]. Given that this training volume reduction is often only 1 week to several weeks pre-competition, our 4–0 month timeframe encompassed both specific competition training and the relative taper, hindering a more nuanced approach to describing a true taper, or clearly identifying which periodization approach was used (classical, reverse, or block periodization plans). However, our findings overall reflected that athletes had relative reductions in training during this multi-month pre-race timeframe, and these relative training reductions coincided with improved race performance. Our findings also concur with prior studies highlighting the importance of total mileage, including easier runs that form an improved running economy [26]. Simultaneously, number of quality sessions was a significant predictor of performance, which is consistent with past work demonstrating the relevance of such training [27]. These findings also coincide with extensive evidence supporting that interval-based training induces preferable adaptations to support improved running performance, such as increased aerobic capacity and muscular power [28]. Our findings overall suggest that varied training approaches were linked with improved endurance performance in a competitive marathon field. Our assessment expands on past work, as we captured data from athletes across a range of levels of running experience. As years of marathon running experience and number of previous marathons were not significant covariates, it appears that endurance training elements have implications for performance across experience levels [9, 22]. Notably, sex was a significant factor, and male participants had higher overall training weekly distance compared with female participants, but female participants had longer running durations and completed more cross-training bouts per week, suggesting sex-based recommendations for training behaviors are warranted.

Incorporating questions specifically designed to distinguish total running sessions and quality sessions in this survey allowed for a more in-depth assessment of the composition of endurance training frequencies for optimizing athletic success. Athletes who included quality sessions in their overall training regimens had better race performance, which signals the importance of training variety as opposed to accumulating running distance alone in training programs. Future research should examine the specific details of quality sessions to obtain more insights into training structure as well as activity intensity through ratings of perceived athlete exertion or physiological metrics. Cross-training among marathon runners in the 4–0 months pre-race was significantly associated with official race times in the current sample, which builds upon prior research that has supported substantial benefits of training outside of running, including cross-training, strength, and conditioning, for endurance athletic performance [12, 29–32]. Physical activity external to dedicated running training undoubtedly contributes to overall training loads imposed on athletes [10, 33, 34], which was corroborated by the weekly running and cross-training sessions interactions. Our survey assessment of cross-training activities did not extricate resistance training from other cross-training frequencies; thus, we cannot determine which specific activities were beneficial for race performance. Future assessments evaluating cross-training features may help elucidate the relationships with endurance performance.

### Pre-race Training Frequency Changes

While overall higher training volumes in 4–0 months pre-race were associated with improved performance, importantly, relatively lower training volumes in 4–0 months versus 12–4 months were associated with best outcomes. Relative reductions in the several-month training mesocycle compared with the overall macrocycle among marathon athletes may reflect a transition from maintaining a training base to reducing training volume to optimize performance [8]. Many marathon training programs implement a transition from general training to race-specific preparations within the 4 months prior to competition that tends to feature higher mileage and much longer singular weekly runs [28, 35–37], and finally a tapering phase with greatly reduced training volume in the weeks leading up to competition [23–25]. The identified reduced training frequency pre-race is in contrast with other training approaches in which athletes markedly increase their training volume just prior to competition (reverse linear periodization) [19, 38]. Reducing training volume leading up to competition by 40–60% in the 5-day to several-week period pre-event has been suggested to have a number of physiologic and psychological benefits for sports performance in endurance athletes [39–41]. Benefits include retaining or even increasing aerobic capacity among endurance athletes [42, 43]; improving muscle power output among endurance athletes, attributed to improved muscle glycogen stores [44, 45]; and improved muscle power related to growth in muscle fibers [44–46], associated with favorable induction of muscle fiber hypertrophy gene expression [39]. In addition to these potential physiologic mechanisms, psychological research has demonstrated improvements in mood state and reduced perceived effort observed concurrently with performance improvements with reduced training volume leading up to competition [43, 47–49]. Gradual reductions in training volume as seen with our findings and the relationship to improved race times appear to be beneficial. Changes in training strategy in the mesocycle compared with the macrocycle support periodization principles.

### Clinical Implications

Our survey findings among athletes competing in a major marathon event highlight key opportunities for education during pre-race phases compared with habitual training. Athletes should be aware that increasing or maintaining training volume in the several months leading up to a major race is not necessarily associated with better performance, particularly for trained/developmental runners included in this sample. Instead, maintaining a training base including steady-state runs and quality sessions before gradually reducing running volume in the race preparatory phases appears, to be more beneficial to performance. These notions combat the idea that adding more volume to any training schedule is always better for individual performance and instead support that 1-year chronic training habits with relatively higher training exposures while allowing for offloading in preparation for competition is preferred. Incorporating cross-training into overall training structures particularly in the 4–0 months pre-race correlated with improved official race performance, further corroborating the importance of training variety in overall programming.

### Limitations and Strengths

The survey-based study design may have led to recall bias; many athletes, however, maintain training logs, and several studies have demonstrated good reliability of long-term recall of physical and sports-related activities among athletes [50, 51]. Given that we did not require that athletes kept training logs for inclusion in this study, the results should be interpreted with some caution. While beyond the capabilities of this study, we acknowledge that other training components (e.g., training intensity distribution [low, moderate, high], activity types, training location, and environment) contribute to performance outcomes. We were unable to distinguish specific cross-training activities (e.g., resistance training, other sport) that may have influenced performance nor continuous data for training or cross-training hours per week, and thus, future research should provide more granular assessments of training. Additional features undoubtedly influence performance, such as nutrition strategies, injury history, and between-session recovery, but were unavailable in the current dataset. Injury history may have also indirectly impacted race performance, given potential secondary training adjustments, though it was beyond the scope of this study to examine this directly. Participants were non-elite athletes, as the majority were in the tier 2 classification “Trained/Developmental”; thus, our findings may not be generalizable to all ability levels. Respondents were primarily white and non-Hispanic/Latinx; the results should be interpreted in the context of the scope of participant demographics. Despite these limitations, our study also had several notable strengths, e.g. collecting data from a large cohort of endurance runners at a major marathon event and prospectively assessing performance.

## Conclusions

Higher overall running training volume and quality sessions in the 12–4 and 4–0 months before the Boston Marathon were associated with better performance. Higher cross-training volume in 4–0 months pre-race was also associated with better performance. Importantly, TFCs marked with a relative taper, including reduced running days in the 4–0 months relative to 12–4 months pre-race, were associated with better performance. Athletes, coaches, and stakeholders should be cognizant of training volume, components, and fluctuations prior to endurance competitions to optimize athlete performance. Stakeholders should additionally be aware of sex-based differences in training and TFCs to tailor management.

## Supplementary Information

Below is the link to the electronic supplementary material.

Supplementary file1 (DOCX 19 KB)

Supplementary file2 (DOCX 19 KB)

Supplementary file3 (DOCX 17 KB)

## Declarations

### Data Availability

Data are available upon reasonable request.
