# Supplementary material for: Training Volume and Training Frequency Changes Associated with Boston Marathon Race Performance
Source: Sports Med. 2025 Sep 6;56(1):243–56. doi: 10.1007/s40279-025-02304-4 (PMC12441744; doi:10.1007/s40279-025-02304-4)
Supplement: Supplementary file 1 — Supplementary file1 (DOCX 19 KB) [file 40279_2025_2304_MOESM1_ESM.docx]

Table S1. Relationships between training behaviors in the 12-4 months pre-race and World Athletics points, accounting for age, sex, and running experience. Linear regression assessing the influence of training behaviors in the 12-4 months pre-race for the 2022 Boston Marathon on World Athletics points.

| **Model Component** | **Outcome Variable** | **Comparison** | **ß Estimate**  **(95% Confidence Interval)** | **t statistic** | **p-value** |
| --- | --- | --- | --- | --- | --- |
| **Predictors** *(Overall Model Fit: R^2^=0.630, p<0.001)* | **Weekly Running Duration** ^a^ | >10 vs. 7.5-10 hours | 23.0 (-9.5, 54.3) | 1.4 | 0.154 |
|  |  | >10 vs. 5-7.5 hours | 26.1 (2.5, 49.7) | 2.2 | 0.033 ^a^ |
|  |  | >10 vs. 2.5-5 hours | 29.6 (-6.2, 65.4) | 1.6 | 0.110 |
|  |  | >10 vs. <2.5 hours | 33.9 (10.5, 78.3) | 2.3 | 0.014 ^a^ |
|  | **Weekly Running Distance** ^a^ | *Continuous* | 2.4 (1.6, 3.3) | 5.6 | <0.001 ^a^ |
|  | **Weekly Running Sessions** ^a^ | *Continuous* | 27.4 (10.3, 44.4) | 3.2 | 0.002 ^a^ |
|  | **Weekly Quality Sessions** ^a^ | *Continuous* | 73.6 (24.6, 122.6) | 2.9 | 0.003 ^a^ |
|  | **Weekly Cross-Training Duration** | >10 vs. 7.5-10 hours | 31.2 (-41.7, 104.0) | 0.8 | 0.410 |
|  |  | >10 vs. 5-7.5 hours | 7.9 (-49.4, 65.3) | 0.09 | 0.926 |
|  |  | >10 vs. 2.5-5 hours | 27.9 (-27.5, 83.2) | 1.0 | 0.318 |
|  |  | >10 vs. <2.5 hours | 40.7 (-28.0, 109.5) | 1.2 | 0.240 |
|  | **Weekly Cross-Training Sessions** | *Continuous* | 2.6 (-2.7, 9.3) | 0.88 | 0.380 |
|  | **Weekly Running Sessions and Weekly Cross-Training Sessions** | *Continuous* | 2.4 (-5.7, 0.9) | 1.4 | 0.161 |
| **Covariates** | **Number of Previous Marathons** | *Continuous* | 0.3 (-0.2, 0.7) | 1.5 | 0.141 |
|  | **Years of Marathon Training** | *Continuous* | -1.9 (-3.5, 6.4) | -0.3 | 0.862 |
|  | **Age** ^a^ | *Continuous* | -7.6 (-8.4, 6.7) | -18.2 | <0.001 ^a^ |
|  | **Sex** ^a^ | *Males vs. Females* | -186.6 (-205.7, -165.3) | -18.3 | <0.001 ^a^ |

^a^ signifies statistical significance at p≤0.050.
