# Supplementary material for: Training Volume and Training Frequency Changes Associated with Boston Marathon Race Performance
Source: Sports Med. 2025 Sep 6;56(1):243–56. doi: 10.1007/s40279-025-02304-4 (PMC12441744; doi:10.1007/s40279-025-02304-4)
Supplement: Supplementary file 2 — Supplementary file2 (DOCX 19 KB) [file 40279_2025_2304_MOESM2_ESM.docx]

Table S2. Relationships between training behaviors in the 4-0 months pre-race and World Athletics points, accounting for age, sex, and running experience. Linear regression assessing the influence of training behaviors in the 4-0 months pre-race for the 2022 Boston Marathon on World Athletics points.

| **Model Component** | **Outcome Variable** | **Comparison** | **ß Estimate**  **(95% Confidence Interval)** | **t statistic** | **p-value** |
| --- | --- | --- | --- | --- | --- |
| **Predictors** *(Overall Model Fit: R^2^=0.633, p<0.001)* | **Weekly Running Duration** ^a^ | >10 vs. 7.5-10 hours | 20.9 (-2.1, 44.5) | 1.3 | 0.199 |
|  |  | >10 vs. 5-7.5 hours | 42.2 (10.6, 77.9) | 2.5 | 0.014 ^a^ |
|  |  | >10 vs. 2.5-5 hours | 41.4 (0.9, 87.1) | 1.9 | 0.050 ^a^ |
|  |  | >10 vs. <2.5 hours | 35.4 (7.1, 63.5) | 2.5 | 0.014 ^a^ |
|  | **Weekly Running Distance** ^a^ | *Continuous* | 2.9 (2.2, 3.5) | 8.6 | <0.001 ^a^ |
|  | **Weekly Running Sessions** ^a^ | *Continuous* | 9.5 (2.5, 38.9) | 2.0 | <0.001 ^a^ |
|  | **Weekly Quality Sessions** ^a^ | *Continuous* | 76.6 (34.5, 115.0) | 3.7 | <0.001 ^a^ |
|  | **Weekly Cross-Training Duration** | >10 vs. 7.5-10 hours | 36.3 (-16.3, 95.3) | 1.1 | 0.276 |
|  |  | >10 vs. 5-7.5 hours | 6.7 (-75.7, 59.1) | 0.1 | 0.982 |
|  |  | >10 vs. 2.5-5 hours | 42.4 (-10.0, 98.1) | 1.5 | 0.125 |
|  |  | >10 vs. <2.5 hours | 68.9 (3.4, 138.7) | 2.0 | 0.047 ^a^ |
|  | **Weekly Cross-Training Sessions** ^a^ | *Continuous* | 7.2 (1.4, 25.3) | 2.3 | 0.023 ^a^ |
|  | **Weekly Running Sessions and Weekly Cross-Training Sessions** | *Continuous* | 4.8 (1.6, 8.1) | 2.9 | 0.003 ^a^ |
| **Covariates** | **Number of Previous Marathons** | *Continuous* | 0.3 (-0.1, 0.7) | 1.5 | 0.137 |
|  | **Years of Marathon Training** | *Continuous* | -1.9 (-3.3, 5.0) | -0.3 | 0.861 |
|  | **Age** ^a^ | *Continuous* | -7.6 (-8.5, -6.8) | -18.2 | <0.001 ^a^ |
|  | **Sex** ^a^ | *Males vs. Females* | -186.4 (-206.6, -166.7) | -18.2 | <0.001 ^a^ |

^a^ signifies statistical significance at p≤0.050.
