# Supplementary material for: Training Volume and Training Frequency Changes Associated with Boston Marathon Race Performance
Source: Sports Med. 2025 Sep 6;56(1):243–56. doi: 10.1007/s40279-025-02304-4 (PMC12441744; doi:10.1007/s40279-025-02304-4)
Supplement: Supplementary file 3 — Supplementary file3 (DOCX 17 KB) [file 40279_2025_2304_MOESM3_ESM.docx]

Table S3. Relationships between training frequency changes from 12-4 and 4-0 months pre-race and World Athletics points, accounting for age, sex, and running experience. Linear regression assessing the influence of training frequency changes from the 12-4 to 4-0 months leading up to the 2022 Boston Marathon on World Athletics points.

| **Model Component** | **Outcome Variable**  **(F-statistic, p-value)** | **Comparison** | **ß Estimate**  **(95% Confidence Interval)** | **t statistic** | **p-value** |
| --- | --- | --- | --- | --- | --- |
| **Predictors** *(Overall Model Fit: R^2^=0.526, p<0.001)* | **Δ Weekly Running Sessions** ^a^ | *Continuous* | 14.6 (1.7, 27.5) | 2.3 | 0.026 ^a^ |
|  | **Δ Weekly Quality Sessions** | *Continuous* | 6.6 (-4.9, 18.2) | 1.1 | 0.259 |
|  | **Δ Weekly Cross-Training Sessions** | *Continuous* | 5.6 (-3.0, 14.3) | 1.3 | 0.203 |
| **Covariates** | **Number of Previous Marathons** | *Continuous* | 0.1 (-0.4, 0.6) | 0.5 | 0.636 |
|  | **Years of Marathon Training** | *Continuous* | -1.6 (-3.2, <0.01) | -2.0 | 0.047 ^a^ |
|  | **Age** ^a^ | *Continuous* | -8.7 (-9.6, -7.8) | -20.3 | <0.001 ^a^ |
|  | **Sex** ^a^ | *Males vs. Females* | -137.6 (-159.5, -115.7) | -12.3) | <0.001 ^a^ |

^a^ signifies statistical significance at p≤0.050.
